# Foliar Illumination Affects the Severity of *Cameraria ohridella* Damage Among Horse Chestnut Species

**DOI:** 10.3390/plants15010086

**Published:** 2025-12-27

**Authors:** Liliya R. Bogoutdinova, Olga V. Shelepova, Helen I. Rostovtseva, Galina N. Raldugina, Ekaterina N. Baranova, Alexander A. Gulevich

**Affiliations:** 1 N.V. Tsitsin Main Botanic Garden, Russian Academy of Sciences, Botanicheskaya 4, Moscow 127276, Russia; shov_gbsad@mail.ru (O.V.S.); greenpro2007@rambler.ru (E.N.B.); 2 All-Russia Institute of Agricultural Biotechnology, Russian Academy of Sciences, Timiryazevskaya 42, Moscow 127550, Russia; 3 K.A. Timiryazev Institute of Plant Physiology, Russian Academy of Sciences, Botanicheskaya 35, Moscow 127276, Russia; ni-fir-titi@mail.ru (H.I.R.); raldugina42@mail.ru (G.N.R.)

**Keywords:** *Aesculus*, ohrid leaf miner, chlorophyll a and b, carotenoids, proline, hyperspectral indices

## Abstract

The influence of crown illumination on leaf damage of horse chestnut species (*Aesculus hippocastanum* L., *Aesculus glabra* Willd, *Aesculus flava* Aiton, *Aesculus pavia* L., *Aesculus* × *carnea* Hayne, *Aesculus parviflora* Walter, *Aesculus chinensis* Bunge) affected by ohrid leaf miner (*Cameraria ohridella* Deschka & Dymić) was studied using some accessions from the arboretum botanical tree collection. *A. hippocastanum*, *A. glabra*, *A. flava* had the lowest chl *a* content in the foliage on the sunlit side of the crown, while in *A. pavia*, *A. parviflora and A. chinensis* this indicator was the highest. The chl a content in the leaves of *A. hippocastanum* and *A. flava* under shaded conditions was 1.3 and 2.4 times higher than in the sunlit part, while in *A. pavia*, *A. parviflora* and *A. chinensis* the chl a content on the shaded side was 1.2, 1.6 and 1.3 times lower. The quantitative content of chl b in the sunlit part of the crown in *A. hippocastanum* and *A. flava* was significantly higher than in the other species. Moreover, while *A. flava* and *A. parviflora* had the highest chl b content in the foliage of the shaded part of the crown, *A. glabra* and *A*. × *carnea* had the lowest. Similarly, differences in proline levels were found in the leaves of different horse chestnut species on the sunny side of the crown. Higher proline levels in less infested species were identified. Water content imbalances due to feeding by leaf miners were most characteristic of the severely affected species. Chlorophyll fluorescence determination revealed high photochemical activity with an effective defense system in resistant species, while non-resistant species exhibited weak defense mechanisms in both sunlight and shade. To assess horse chestnut species the hyperspectral analysis indices (DSWI and SIPI) were also successfully applied. Changes in chl a and chl b content, proline levels, and leaf water-holding properties can be used to assess the resistance of horse chestnut species using classical physiological and biochemical methods. Hyperspectral analysis indices (DSWI and SIPI) can also be successfully applied.

## 1. Introduction

The horse chestnut is a very common ornamental tree, widely used in landscaping in many cities around the world. Its popularity is due to its beautiful blooms, tolerance to urban growth factors, and longevity.

Species of the genus *Aesculus* vary in susceptibility to ohrid leaf miner (*Cameraria ohridella* Deschka & Dimič), with the horse chestnut *Aesculus hippocastanum* L. being the primary host of this pest [1]. The feeding of the larvae of this leaf miner and the gnawing of passages in the leaf tissues causes premature leaf fall of *Aesculus hippocastanum* L. [2]. A number of other *Aesculus* species are also susceptible to *C. ohridella*, such as *Aesculus turbinata* Blume, *Aesculus sylvatica* Bartram, *Aesculus pavia* L., *Aesculus flava* Aiton, and *Aesculus glabra* Wild [3,4,5]. Horse chestnuts species resistant to the ohrid leaf miner include *Aesculus indica* (Wall. ex Camb.) Hook, *Aesculus californica* (Spach) Nutt., *Aesculus parviflora* Walter, *Aesculus assamica* Griff. [6,7], *Aesculus* × *carnea* Hayne, *Aesculus parviflora* and *Aesculus chinensis* [5,8].

Changes in pigment levels, particularly the levels of chlorophylls *a* and *b* and their ratios, are crucial for understanding the mechanisms of plant adaptation to stress. A decrease in the quantitative content of photosynthetic pigments under various stress conditions can reduce the rate of carbon assimilation and the formation of photosynthetic products [9]. Chlorophyll fluorescence is known to be used as an indicator of physiological processes [10]. Carotenoids have antioxidant properties and play an important role in plant adaptation to adverse environmental factors [11]. At higher light levels or in response to biotic stress, carotenoid concentrations may increase, as zeaxanthin, formed in the xanthophyll cycle, is synthesized in plants to reduce the negative impact of ROS [12].

Carbohydrates produced during photosynthesis are an essential and attractive nutrient for phytophagous insects [13], and plants containing higher amounts of carbohydrates are more susceptible to pests, including the horse chestnut leaf miner [14]. Thus, the maximum amount of carbohydrates was found in the leaves of three horse chestnut species: *A. hippocastanum*, *A. glabra*, and *A. flava*, which are most susceptible to the orchid leaf miner—54.1; 46.3; and 42.7%, respectively. The lowest amount of carbohydrates in the leaves of such a leaf miner-resistant horse chestnut species as *A. chinensis* was observed [5]. It has been suggested that pests may interact with plants through visual stimuli [15,16]. Green leaf blades of host plants are readily colonized by herbivorous insects, whose larvae and caterpillars feed on leaf tissue [17]. Female horse chestnut leaf miners lay eggs on leaves with a large proportion of green surface because such leaves have more food for the growth and development of larvae [18].

Tree canopies exhibit different illumination patterns, and leaves developing under these conditions exhibit differences in morphology and biochemistry [19]. Thus, leaf biomass and area are positively correlated with daily photon irradiance [20].

The water balance of infected leaves can change depending on the intensity of infection [21]. Biotic stress also alters proline content, for example, in *Ulmus* [22] and *Populus* [23]. In the aboveground parts of plants, proline content often serves as a marker of osmotic stress after insect damage, for example, in *Melolontha melolontha* [24], in which its content was shown to increase during the feeding of insect larvae. Additionally, higher proline levels increase the NADP+/NADPH ratio, enhancing the synthesis of phenolic compounds directly involved in plant defense [25].

Methods for remote monitoring of plant health are currently being actively developed. Important areas of this development are based on identifying relationships between plant parameters and their optical characteristics. Key methods of optical remote monitoring include measuring chlorophyll fluorescence, RGB imaging, and analyzing the spectral characteristics of light reflected by plants [26]. This method is based on differences in reflectivity, which manifests itself in variability in the optical properties of plants [27,28]. The chlorophyll and photochemical reflectance indices, as well as water content, appear promising for this purpose. The chlorophyll index is closely related to the chlorophyll content of leaf tissues and can serve as a measure of the capacity of the photosynthetic apparatus and the potential ability of the plant to absorb solar radiation [29]. These indices have found application in characterizing stress conditions caused by mineral nutrient and moisture deficiencies [28,30].

Thus, we believe that the study of physiological and biochemical parameters allows us to assess the condition of horse chestnut species affected by the leaf miner, differing in their resistance to the pest (*Aesculus hippocastanum* L., *Aesculus glabra* Willd, *Aesculus flava* Aiton, *Aesculus pavia* L., *Aesculus* × *carnea* Hayne, *Aesculus parviflora* Walter, *Aesculus chinensis* Bunge). It was also necessary to find out that the illumination of the crown can influence both the number of pests and the physiological state of plants affected by this pest. This factor may be the reason for the decrease in the decorative properties of plants in urban environments. Thus, the aim of the study was to evaluate the influence of crown sunny illumination on changes in biochemical processes associated with foliage damage by the ohrid leaf miner in various horse chestnut species.

## 2. Results

### 2.1. Determination of the Second Generation Population of C. ohridella

**Figure 1:**
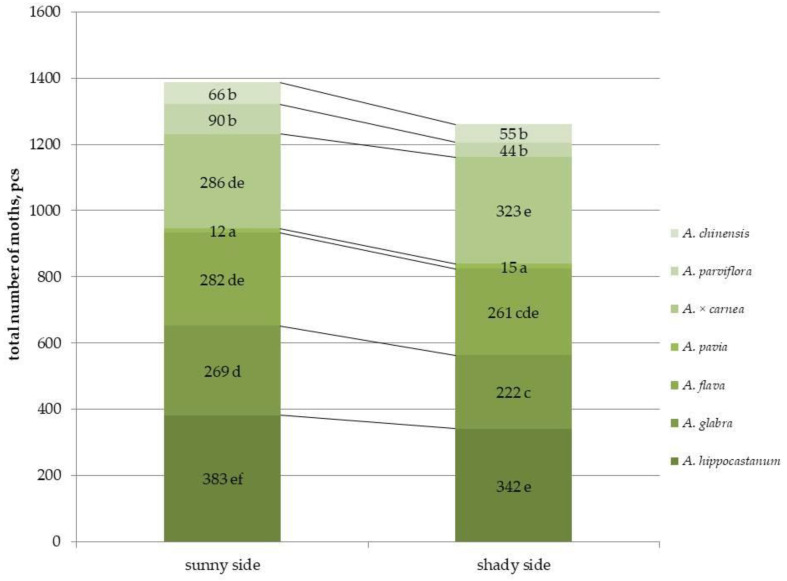
Total abundance of second-generation *C. ohridella* moths on leaves of different horse chestnut species, under conditions of varying sunlit illumination. Values are presented as mean ± standard error at α = 0.05 according to ANOVA tests. Letters indicate significant differences were determined (α = 0.05).

The study revealed differences in the abundance of the horse chestnut leaf miner on different horse chestnut species in the second generation (Figure 1 and Figure S1).

Differences in the number of leaf miners in traps on parts of the crown with different illumination were observed only for a few species, namely *A. hippocastanum*, *A. glabra*, and *A. parviflora*. These species showed significant reductions in moth abundance by 1.1, 1.2, and 2 times, respectively. However, no differences were found between the sunlit and shaded parts of the crown in other species. Among all species, the highest number of leaf miners was observed in *A. hippocastanum* (an average of 382 males per trap), which was 30 times more than *A. pavia*, which had the lowest number of moths per trap (an average of 12 males per trap). *A. parviflora* and *A. chinensis* also showed the lowest number of leaf miners in traps, 90 and 66 moths, respectively. Thus, the most affected species, regardless of crown illumination, were *A. hippocastanum*; *A. glabra*; *A. flava* and *A. × carnea*, and the least affected species included *A. pavia*; *A. parviflora* and *A. chinensis*. The results obtained were confirmed by the number of mines per leaf (Figure 1 and Figure S1). For example, in *A. hippocastanum*, the number of mines reached 78, while in *A. parviflora* and *A. chinensis*, virtually no mines were found on the leaf (1 mine per leaf) (Figure S2). Duncan’s test for pairwise comparisons revealed statistically significant differences between plants species damaged by leaf miner (Table S1).

**Figure 2:**
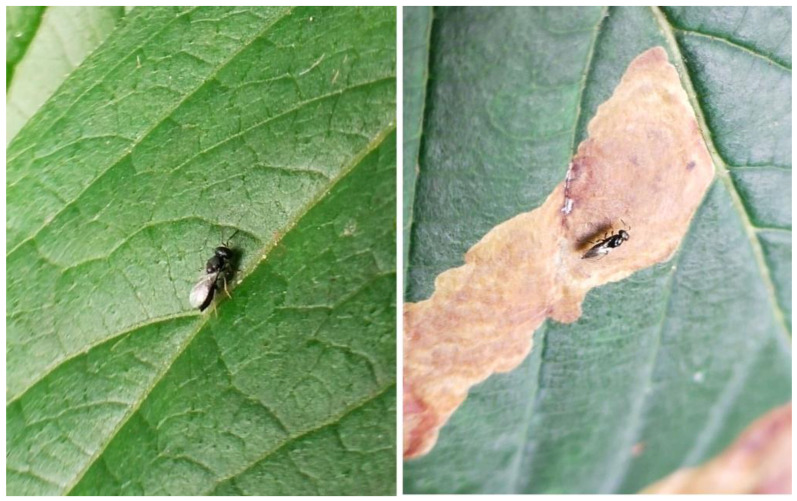
Parasitic wasps (*Pteromalidae* sp.) on the *A. hippocastanum* leaf surface (**left**) and on the mines (**right**), formed by the caterpillar of the ohrid leaf miner (*C*. *ohridella*).

Parasitic wasps were found on the leaves in the most affected horse chestnut species, *A*. *hippocastanum*, at all stages of damage (Figure 2).

### 2.2. Determination of Photosynthetic Pigment Content

**Figure 3:**
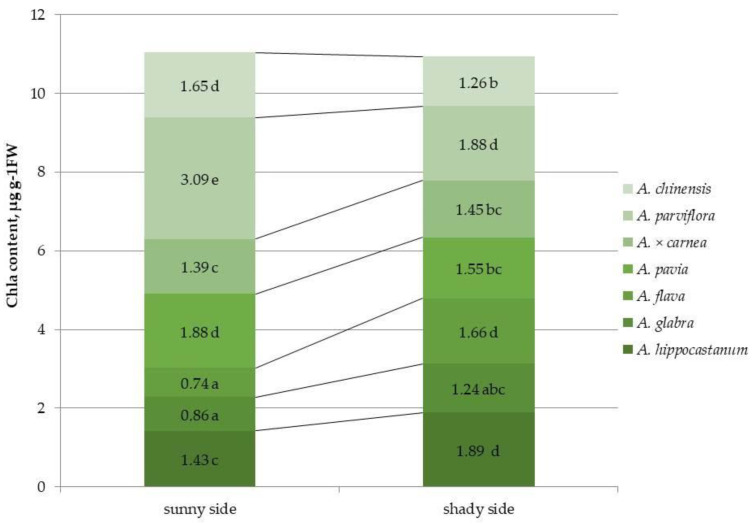
Chlorophyll a (chl *a*) content in leaves of different horse chestnut species affected by *C*. *ohridella* under varying foliage illumination conditions. Values are presented as mean ± standard error at α = 0.05 according to ANOVA tests. Letters indicate significant differences were determined (α = 0.05).

Different horse chestnut species have been evaluated for their photosynthetic pigment content under different light conditions (Figure 3 and Figure S3). The species most affected by the leaf miner, namely *A. hippocastanum*, *A. glabra*, and *A. flava*, had the lowest quantitative content of chl *a* on the sunlit side, while the least affected species, *A. pavia*, *A. parviflora*, and *A. chinensis*, had the highest value. In *A*. × *carnea*, the value of the indicator was average. It was found that the chl *a* content in *A. hippocastanum* and *A. flava* under shaded conditions was 1.3 and 2.4 times higher than in the sunlit part of crown, while in *A. pavia*, *A. parviflora*, and *A. chinensis*, the chl *a* content was 1.2, 1.6 and 1.3 times less than on the sunny side.

**Figure 4:**
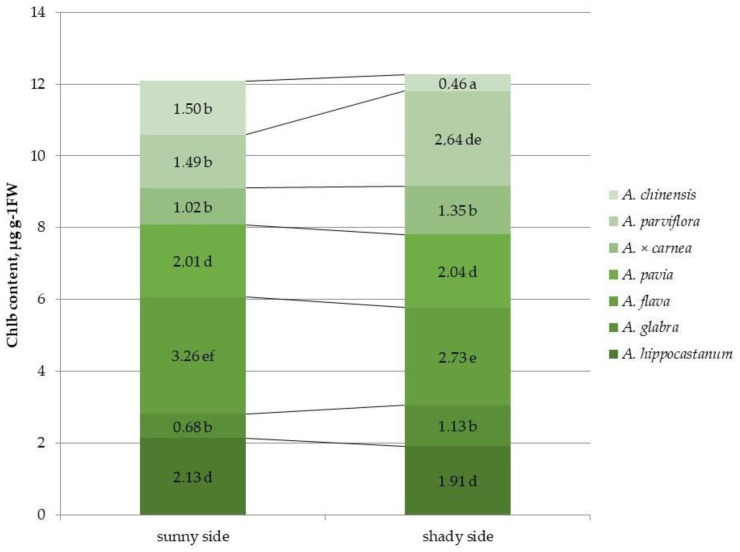
Chlorophyll b (chl *b*) content in leaves of different horse chestnut species affected by *C*. *ohridella* under varying foliage illumination conditions. Values are presented as mean ± standard error at α = 0.05 according to ANOVA tests. Letters indicate significant differences were determined (α = 0.05).

In contrast to the chl *a* content, the chl *b* content in the heavily affected species, *A. hippocastanum* and *A. flava,* was significantly higher than in the other species in the illuminated part of the crown. (Figure 4 and Figure S4). Moreover, in *A*. *flava* and *A*. *parviflora*, the highest amount of chl *b* was noted in the leaves of the shaded part of the crown, and in *A*. *glabra* and *A*. × *carnea* this indicator had the lowest value. In contrast to chl *a*, an increase in the concentration of chl *b* under shading was found only in *A. parviflora*. The remaining species showed either a reliable decrease in this indicator, as in *A. hippocastanum*, *A*. *flava* and *A*. *chinensis* (by 1.1, 1.2 and 3.3 times, respectively), or the content of chl *b* did not differ depending on the illumination of the crown, as in *A. glabra*, *A. pavia* and *A*. × *carnea*.

**Figure 5:**
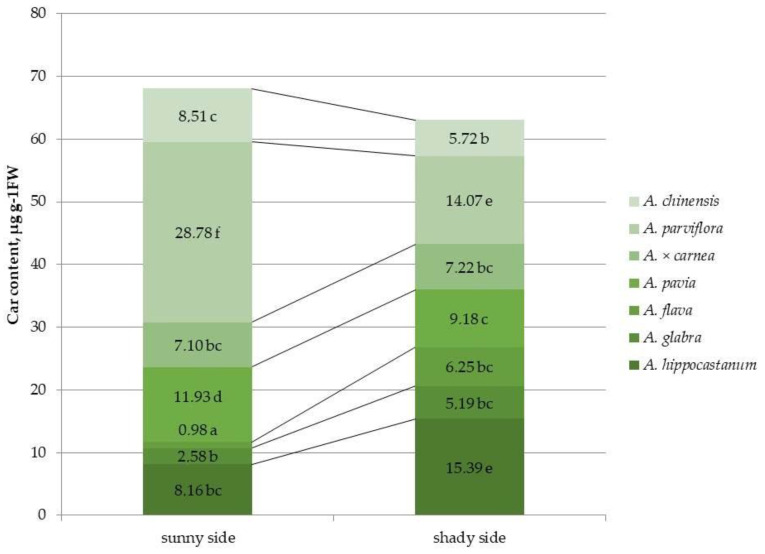
Carotenoid (car) content in leaves of different horse chestnut species affected by *C*. *ohridella* under varying foliage illumination conditions. Values are presented as mean ± standard error at α = 0.05 according to ANOVA tests. Letters indicate significant differences were determined (α = 0.05).

The carotenoid content in the species *A. hippocastanum*, *A. glabra* and *A. flava* was 1.9, 2.01 and 3 times higher, respectively, under shaded crown conditions compared to the sunlit side (Figure 5 and Figure S5). The remaining species, except *A*. × *carnea*, showed a significant decrease in this parameter under shaded conditions. The carotenoid content of *A*. *parviflora* in illuminated canopy conditions was the highest and 11.1 and 8.6 times higher than the lowest values in *A. glabra* and *A. flava*, respectively. *A. parviflora* also had the highest carotenoid concentration under shaded conditions, but in that case, *A*. *hippocastanum* can also be noted with a similar value for this parameter.

### 2.3. Determination of Proline Content

**Figure 6:**
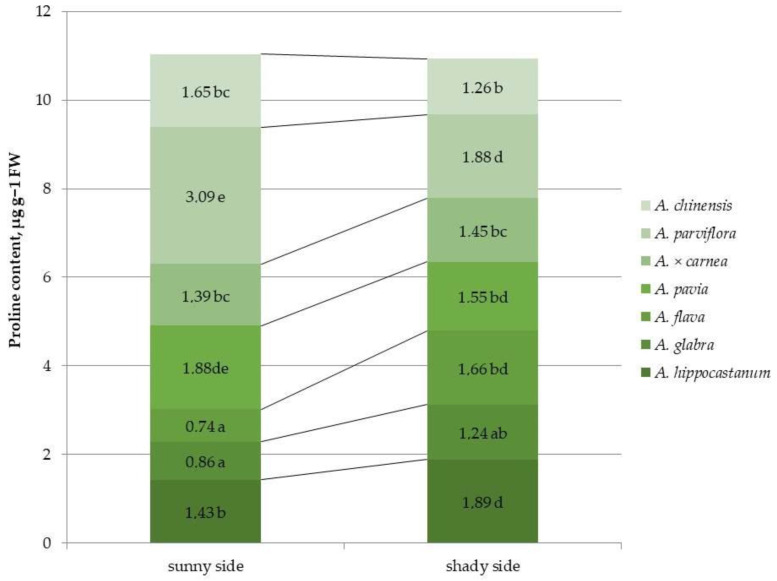
Proline content in leaves of different horse chestnut species affected by *C*. *ohridella* under varying foliage illumination conditions. Values are presented as mean ± standard error at α = 0.05, according to ANOVA tests. Letters indicate significant differences were determined (α = 0.05).

Differences in proline content were observed between horse chestnut species (Figure 6 and Figure S6).

Among all of the species, the highest proline content under sunlit crown conditions was shown in *A. pavia* and *A. parviflora*, and the lowest in *A. glabra* and *A. flava*. Under shaded canopy conditions, *A. parviflora* also showed the highest proline content, which was approximately equal to that of *A. hippocastanum*. A significant increase in proline content under shaded conditions was found in the species *A. hippocastanum* and *A. flava* (1.3 and 2.24 times, respectively). The proline content in leaves decreased in species, such as *A. parviflora*, *A. pavia* and *A. chinensis* (by 1.6, 1.2, 1.3 times, respectively), under shaded conditions compared to the sunlit side.

### 2.4. Determination of Dry Leaf Biomass

**Figure 7:**
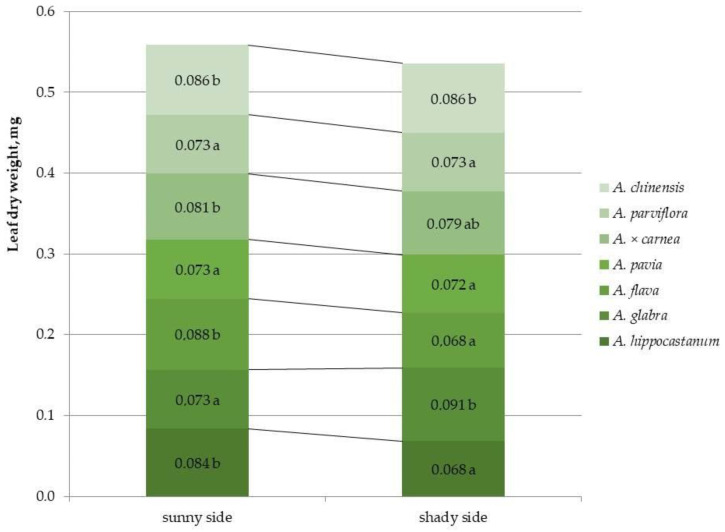
Dry biomass of leaves of different horse chestnut species affected by *C*. *ohridella* under varying foliage illumination conditions. Values are presented as mean ± standard error at α = 0.05 according to ANOVA tests. Letters indicate significant differences were determined (α = 0.05).

For most species, crown illumination did not affect the dry leaf biomass index, with the exception of *A. hippocastanum* and *A. flava*, which showed a decrease in this index under shaded conditions (by 1.2 and 1.3 times, respectively) and *A. glabra*, for which the dry biomass value under crown shaded conditions increased by 1.2 times (Figure 7 and Figure S7).

Among all species, the highest value of this characteristic under sunlit crown conditions was shown by *A. flava*, and under shaded crown conditions the highest index was noted for *A. glabra, A. × carnea and A. chinensis.*

### 2.5. Detection of Water Content in Leaves of Different Horse Chestnut Species

**Figure 8:**
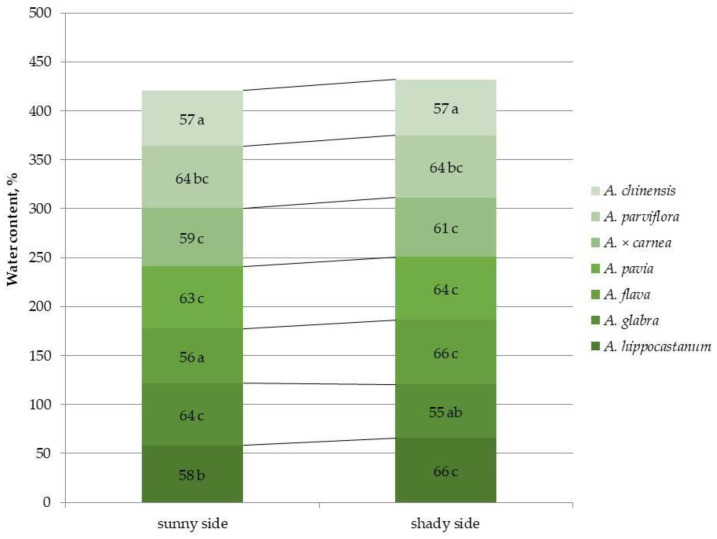
Leaf water content in different horse chestnut species affected by *C*. *ohridella* under varying foliage illumination conditions. Values are presented as mean ± standard error at α = 0.05, according to ANOVA tests. Letters indicate significant differences were determined (α = 0.05).

An important parameter characterizing the water status under abiotic stress is the water content of plant tissues. Leaf water content was calculated based on the fresh and dry biomass of leaves (Figure 8 and Figure S8).

Among all horse chestnut species *A. flava* and *A. chinensis* exhibited the lowest water content on the sunny side of the crown. The highest leaf water content under shaded conditions was found in *A. hippocastanum* and *A. flava*. No significant differences were found in this indicator for most species under different lighting conditions. In the highly affected species *A. hippocastanum* and *A. flava*, a 1.1-fold increase in leaf water content was observed under shaded conditions, while *A. glabra* showed a 1.2-fold decrease in this parameter under shaded conditions.

A strong correlation between the number of mines and the number of moths in traps (r = 0.8) was revealed the potential relationship between these parameters. On the other hand, we have shown a negative correlation between the number of leaf miners in traps and chl a content (r = −0.3). Chl a content decreased accordingly with plant damage caused by leaf miners. A weak negative correlation was also found between the chl b content and numbers of leaf mines (r = −0.3). In addition, there were no relationships between chl b and the number of leaf miners in traps and plant dry biomass, accordingly. A negative correlation was found between the number of mines, leaf miner numbers in traps and the quantitative carotenoid content (r = −0.2 and −0.3) or water content (r = −0.18 and −0.1) (Table S2).

### 2.6. Determination of Hyperspectral Indicators

Using hyperspectral analysis, some of the most common indices reflecting the physiological states of the plant were studied (Table 1).

To assess the possibilities of analyzing photographs obtained with an optical camera under the same lighting conditions, an analysis was carried out of the RSB indices of images of the surface of leaves of various types of horse chestnut taken with an optical camera under artificial halogen lighting.

Thus, no significant difference was found between horse chestnut species in the NDWI index, regardless of crown illumination. However, changes were already detected between the species in the DSWI index. This index decreased in *A. glabra* (from 3.284 to 2.866) and *A. pavia* (from 2.97 to 2.4160). Nevertheless, in one of the least affected species, specifically *A. chinensis*, the index value increased from 2.786 to 3.364 under shaded conditions. The highest DSWI value was found in *A. chinensis* under crown shaded conditions and amounted to 3.364, while the lowest index was shown in *A. parviflora* (1.888) under shaded conditions.

One of the key indicators characterizing the efficiency of the plant photosynthetic apparatus is the chlorophyll index. A decrease in the SIPI photochemical index in some affected horse chestnut species was found under conditions of reduced foliage illumination, from 1.403 to 1.218 in *A. glabra* and from 1.365 to 1.248 in *A. pavia*. This index increased with crown shading only in two species, *A*. × *carnea* (from 1.157 to 1.211) and *A. chinensis* (from 1.331 to 1.418).

### 2.7. Determination of Leaf Chlorophyll Fluorescence

Based on chlorophyll fluorescence measurements, a comparative analysis can be conducted and a clear ranking of the tolerance of various horse chestnut species to light stress can be developed (Table 2 and Table 3). All studied species exhibited characteristics of photoinhibition to varying degrees, as their key indicator of photosystem health, Fv/Fm, was below the optimal level. However, their ability to cope with this stress varied significantly.

*A. chinensis* and *A. parviflora* proved to be the most adapted and efficient species. Their photosynthetic apparatus demonstrates a balanced balance: in sunlight, they exhibit the highest values of both potential efficiency (Fv/Fm) and actual productivity (ETR). This success is due to powerful defense mechanisms—the ability to actively dissipate excess light energy as heat (high NPQ and Y(NPQ) values), which minimizes damage (their Y(NO) value is the lowest). It is important that these species also maintain leadership in shady conditions, which indicates their high plasticity and ability to work productively in different light conditions.

The group of horse chestnut species with moderate tolerance to leaf miner includes *A.* × *carnea*, *A. pavia* and *A. glabra*. Their photosynthetic apparatus is functional but experiences significant stress. Their defense systems are moderately developed, resulting in moderate levels of damage. When exposed to shade, their productivity significantly decreases, indicating a narrower range of adaptation compared to the leading species.

The least resilient and most vulnerable species were *A. flava* and, especially, *A*. *hippocastanum*. The photosynthetic apparatus of these species is severely impaired. They are unable to effectively defend themselves against excess light, as evidenced by the weakest photoprotection indices (NPQ, Y(NPQ)). Consequently, they suffer the greatest losses—demonstrating an extremely high proportion of uselessly dissipated energy (Y(NO)), which not only is not used for photosynthesis but also aggravates damage. This is reflected in very low productivity both in the light and, critically, in the shade. Particularly significant is the sharp decline in *A. hippocastanum* activity in the shade, indicating the inability of its photosystem to function under limited energy conditions.

## 3. Discussion

The crowns of mature trees can have both shaded and sun-exposed areas, creating different living conditions for organisms [19]. Larvae of leaf-eating pests (*Spodoptera frugiperda* and *Spodoptera litura*) are exposed to sunlight during feeding. Light intensity affects only the development of male leaf-eating insect larvae, while changes in the development of both male and female larvae have been demonstrated in *Ostrinia furnacalis* and *Chilo suppressalis*. Developmental stages of these insect species were significantly delayed in complete darkness [31]. For example, we previously showed that crown sunlight illumination in *A. hippocastanum* affected the abundance and population density of *C. ohridella* during the growing season [32]. As a result, the highest pest abundance was observed on the sunlit side of the tree crown, and the lowest on the shaded side. This may be due to the larger food supply, both due to the high carbohydrate content and the large cell surface area of the palisade parenchyma and lower epidermis. In a study of horse chestnut species collection, a higher number of miners were also found in traps in the illuminated part of the *A. hippocastanum* crown (an average of 382 males per trap). This is consistent with our previous studies conducted in 2024 on this collection of horse chestnut species [5]. In a study of a collection of horse chestnut species, a higher number of miners were also found in traps of *A. hippocastanum* on the sunlit side of the crown. Fewer moths were also found on the shaded side of the crown for two species, *A. glabra* and *A. parviflora*, while for other species, pest abundance was equal on both the sunlit and shaded sides. It was also shown that the species most affected, regardless of crown sunlight illumination, were *A. hippocastanum*, A. glabra, *A. flava* and *A*. × *carnea*, while the least affected species included *A. pavia*, *A. parviflora* and *A. chinensis*.

The chemical composition of host plant leaves significantly influences insect behavior [14,33]. Moreover, the chemical composition itself can vary under different illumination conditions [19]. The amount of pigments in the leaves determines the color of these organs and serves as a visual signal that facilitates the detection of the host plant by adult *Lepidoptera* [16]. It was found that leaf mining by larvae causes changes in the volatile compounds of the affected leaves, which prevents other individuals from laying eggs on the same leaves, which may facilitate the search for new green leaves by other females of the horse-chestnut leaf miner [34]. Thus, it was previously found that the total chlorophyll content in the leaves of *A*. × *neglecta*, which is completely resistant to *C. ohridella*, was significantly higher than that of *A. turbinata*, which is infested by this pest [14]. Similarly, higher chlorophyll levels in *A. hippocastanum* leaves coincided with a period when it was less colonized by the horse chestnut leaf miner. *A. hippocastanum*, *A. glabra*, and *A. flava* had the lowest quantitative content of chl *a* on the sunlit side, and in the least affected species by the leaf miner, *A. pavia*, *A. parviflora* and *A. chinensis*, this indicator was the highest. Under shaded conditions, *A. hippocastanum* and *A. flava* showed an increased content of chl *a* compared to the illuminated part, while in the less affected species, *A. pavia*, *A. parviflora* and *A. chinensis*, the content of chl *a* was lower. However, the opposite situation was observed for the chl *b*: in *A. hippocastanum* and *A. flava*, in the sunlit crown part, the value of this indicator was significantly higher than in other species. This fact may be due to the fact that the changes in the chlorophyll content in the leaves discovered in the present study may be associated with a disruption in the biosynthesis or accelerated degradation of chlorophyll a and the accumulation of chlorophyll b [35]. Differences in the amount of carotenoids between horse chestnut species have also been shown previously. Thus, the carotenoid content in the leaves of *A*. × *neglecta* was significantly higher than in the susceptible species *A. turbinata* [14]. In our study, the highest carotenoid content was also shown in the resistant species *A. parviflora*, regardless of lighting conditions.

Moreover, a study of chlorophyll fluorescence indices revealed two opposing types of adaptation. Resilient species successfully combine high photochemical activity with an effective defense system, allowing them to minimize damage and maintain productivity under various conditions. Conversely, susceptible species have weak defense mechanisms; their photosynthetic apparatus operates inefficiently, and is chronically damaged, making them vulnerable in both sunlight and shade.

Biotic stress alters the quantitative content of not only pigments, but also proline and phenolic compounds, for example, in *Fabaceae* species [23]. It was shown that the amount of proline doubled in leaves after aphid (*Chaitophorus nassonowi*) infestation [36]. Under shade conditions, soybean plants showed an increase in total proline and some antioxidant content, indicating increased stress [37]. Lackner, S. and co-authors [24] showed that leaves with higher proline levels are preferred by *Lymantria dispar*, while, other studies have not found an increase in proline levels in *Populus tremula* after infestation by the sponge moth [36]. The lowest proline content was observed in species, such as *A. parviflora*, *A. pavia* and *A. chinensis*, which may be due to lower stress conditions and, accordingly, lower proline synthesis. In our study, a significant increase in proline content under shaded conditions was found in the most affected species *A. hippocastanum* and *A*. *flava*, which is consistent with another study [24]. Under shaded conditions, proline content in leaves decreased in the least populated species *A. parviflora*, *A. pavia*, and *A. chinensis*. The highest proline content, regardless of light conditions, among all species was observed in *A. parviflora*. This may be due to the fact that higher proline levels enhance the synthesis of phenolic compounds that are involved in plant defense [38,39]. Despite this, a positive effect of proline on the development of populations of some insects, such as aphids, has been shown [36].

Hydraulic conductivity, plant water status, and stomatal conductance underlie plant–insect interactions [39]. Changes in xylem fibers [40] and changes in hydraulic conductivity can also be associated with insect damage [41]. In *A. chinensis*, the lowest water content was found on the sunny side of the crown compared to other species. As a result, it can be noted that heavily infested *A. hippocastanum* and *A. flava* showed an increase in leaf water content under shaded conditions. Thus, no significant differences were shown between horse chestnut species under lighting conditions, but they were shown under shading conditions. This may be due to the fact that leaf miners disrupt the water-holding properties of the leaf by feeding on both the surface and deeper living tissues, which, in turn, leads to an increase in plant transpiration [42]. The decrease in raw biomass and changes in water content in general are due to a disruption in water exchange [43]. For example, leaves on the sunny side of the canopy had higher fresh weight per unit area, as well as dry weight, leaf blade thickness, vein and stomatal density, net photosynthetic rate, and stomata conductance compared to leaves on the shaded crown side [44,45]. The hydraulic conductance of leaves exposed to sunlight increased almost fourfold compared to the minimum values in the shadow [45]. The present planting is located within a dense arboretum, where other plants of the damaged species are absent. However, when studying open spaces, in addition to the characteristics of the species, the nature of the foliage lighting, and ecological and climatic conditions, it is necessary to take into account that adults overcome ranges from 7.9 to 12.6 km on average [46] and environmental factors, such as tree position relative to wind directions, may have an impact.

Hyperspectral imaging sensors are effective methods for detecting changes in plant health [47]. Changes in reflectance are caused by the biophysical and biochemical properties of plant tissue. Under stress, tissue color, leaf shape, and transpiration rate can change, as can the interaction of solar radiation with plants [48]. NDVI is a dimensionless index that characterizes vegetation as the difference between visible and near-infrared radiation. This index is one of the most commonly used for monitoring vegetation dynamics at regional and global scales [49]. However, in our case, the NDVI index did not reveal significant differences between horse chestnut species, making it unsuitable for detailed classification of tree species by water content.

The next index analyzed is the DSWI, which characterizes plant stress caused by water deficit and damage [50]. The sensitivity of the DSWI index to tree species differentiation was demonstrated in a previous study [50] on conifers (pine, spruce) and deciduous trees (hornbeam, oak). Our study also demonstrated differences between horse chestnut species, with the highest index demonstrated by most species and the lowest by *A*. × *carnea* and *A. parviflora*, regardless of lighting conditions. This index allowed us to distinguish between plant species regardless of crown lighting conditions.

The last of the three indices studied was the SIPI (structure-insensitive pigment index), which reflects the efficiency with which a plant can utilize incoming light for photosynthesis. Moreover, the greatest differences were found in the illuminated part of the crown. SIPI was highest in some severely affected horse chestnut species (*A. glabra*, *A. flava*, and *A. pavia*), while the lowest was found in *A. hippocastanum*, *A. chinensis*, *A*. × *carnea*, and *A. parviflora*. Therefore, this index can be used to evaluate plants, but not in shaded conditions, where the differences would be insignificant.

A high negative correlation was observed between the DSWI and SIPI coefficients and the chl *a* content in the sunlit part of the crown (r = −0.5 and below). However, a high positive correlation was found between the Fv/Fm and NPQ indices and the chl *a* content (r = 0.5 and above). Under shaded conditions, a negative correlation was found between the chl *a* content and the hyperspectral analysis indices and chlorophyll fluorescence indices (Table S1).

Interestingly, a weak positive correlation (r = 0.2–0.4) was observed between the DSWI, SIPI, and Y(NPQ) coefficients and the chl *b* content in the illuminated part of the crown. In all other cases, a weak negative correlation (r = −0.06–0.5) was found between the chl *b* content and the hyperspectral analysis indices and chlorophyll fluorescence indices (Table S3).

## 4. Materials and Methods

### 4.1. Place of Research and Plant Material

Collection of *Aesculus* species (*Aesculus hippocastanum* L., *Aesculus glabra* Willd, *Aesculus flava* Aiton, *Aesculus pavia* L., *Aesculus × carnea* Hayne, *Aesculus parviflora* Walter, *Aesculus chinensis* Bunge) in the arboretum territory in the Main Botanical Garden (MBG) of the Russian Academy of Sciences (RAS) in Moscow (55.838° N, 37.588° E) was analyzed [51,52,53]. The *Aesculus* species trees in the arboretum’s collection range in age from 10 to 80 years old, so we selected freestanding trees with a clearly defined, open space on the south side to avoid shading. The collection is located in the center of a mixed forest, dominated by common oak and separated from other stands of horse chestnut and other species known to be susceptible to the ohrid leaf miner. The foliage beneath the trees is not cleared, and no treatments are carried out due to a ban on chemical treatments. Three trees of each of the seven species affected by the horse chestnut leaf miner were selected for the study (*Aesculus chinensis* Bunge). Only two young trees, relatively recently planted in the arboretum’s collection, were used in the experiments. In the year 2024, monitoring of plants was carried out, including an analysis of the number of the second generation of the horse chestnut leaf miner.

### 4.2. Estimation of Horse Chestnut Miner Abundance Using Pheromone Traps

Delta sticky pheromone traps with dispensers impregnated with the synthesized sex pheromone of female moths (Pheromon, Moscow, Russia) were hung on horse chestnut trees in different parts of the crown. The second generation of adults in the foliage of the horse chestnut collection was counted from July to August. Traps in three biological replicates on two to three trees were attached to horizontal branches of the outer part of the horse chestnut crown at a height of 1.5–2 m from the ground [31]. Sticky plates in the traps were extracted once.

Pheromone insect counting traps were attached to the branches of three trees of the each species being studied (one trap on each of the three trees of the same species, one on the sunny and one on the shaded side). One trap was hung 1.5–2 m above the ground, and the other on the opposite side of the tree. The distance between the trees was 2–3 m. In this study, the abundance of the generation of males of the horse chestnut leaf miner was investigated. Sticky plates in the traps were extracted once. A schematic representation of the arrangement of traps for capturing adult male ohrid leaf miner moths in the crowns of horse chestnut trees used in this study reflects the choice of location based on spatial orientation relative to cardinal directions to account for solar illumination when selecting isolated trees (Figure S9).

### 4.3. Morphometric Indicators

To determine the water content of leaves of seven horse chestnut species affected by *C. ohridella*, averaged 0.2 g samples were collected. Leaf biomass was determined gravimetrically using the Sartorius Analytical A200S (Sartorius, Göttingen, Germany). To determine dry biomass, leaves were dried at 65 °C to constant weight. Each experimental variant was performed in three biological replicates.

*RWC = [(FW − DW)/FW]* × 100%

where RWC—relative water content (%), *FW* and *DW*—fresh and dry biomass of root or shoot part of seedling, respectively (mg).

### 4.4. Determination of Leaf Pigment Content

The photosynthetic pigment content (chlorophylls a (chl *a*), and b (chl *b*) and carotenoids (Car)) were determined by extracting pigments from leaves with 96% ethyl alcohol [54]. The degree of solution absorption (optical density) for chlorophylls a and b, and carotenoids was determined using Genesys 20 spectrophotometer (ThermoScientific, Waltham, MA, USA) at a wavelength of 665, 649 and 471 nm, respectively. Each experimental variant was performed in two analytical and three biological replicates. The pigment content (µg g^−1^ FW) was calculated by the formulas [55]:

Cchl a = 13.70D_665_ − 5.76 D_649_;

Cchl b = 25.80 D_649_ − 7.60 D_665_;

Ccar = (1000D_471_ × 2.13Cchl a − 97.64Cchl b)/209

A = C × V/1000 × n

where: C—pigment concentrations; D—optical density; V—extract volume; and n—leaf fresh weight [56].

### 4.5. Detection of Leaf Proline Content

Extraction and determination of free proline were performed according to Bates et al. (1973) with minor modifications [57]. A weighed sample of plant material weighing 0.1 g was triturated in liquid nitrogen. The ground material was transferred into test tubes and filled with 4 mL of distilled water. Test tubes with weighed samples were brought to a boil three times, cooling each time. The resulting extract was filtered into measuring tubes. The resulting extract was adjusted to the desired volume (6–7 mL) and then used for analysis. Tubes with 1 mL extract, 1 mL glacial acetic acid, 1 mL ninhydrin reagent (1.25 g ninhydrin, 20 mL 6M H_3_PO_4_, 30 mL CH_3_COOH) were incubated for 1 h in a boiling water bath. Instead of the extract, 1 mL of distilled water was added to the control sample. The optical density of the obtained colored solutions was measured on a PD-303 spectrophotometer (Apel, Saitama, Japan) against the control at a wavelength of 520 nm. Each experimental variant was performed in two analytical and three biological replicates. The proline content was calculated using the calibration curve according to the formula:

C = Ek × V/(W × 1000),

where: C—proline concentration, µM g^−1^ fresh weight, E—optical density, k—coefficient calculated from the calibration curve, V—extract volume, mL, W—sample weight, g.

### 4.6. Chlorophyll Fluorescence Characteristics

Chlorophyll fluorescence characteristics were determined on the leaf surface. Seedlings were kept in a dusky place for 30 min before the measurements. Modulated fluorescence was recorded with a portable chlorophyll fluorometer, the blue version Junior-PAM (Heinz Walz GmbH, Effeltrich, Germany). This version is emitted between 400 and 500 nm with a maximum around 445 nm and is equipped with a far-red LED with maximal emission around 745 nm and an emission range from 675 to 800 nm. The least fluorescence value (*F_0_*) was measured for 30 min in dusky-adapted leaves using light of <0.1 µM photons/(m^−2^ s^−1^), the maximum fluorescence value (*Fm*) was recorded at 1000 µM photons/(m^−2^ s^−1^) photosynthetic photon flux density in the same leaves. The intensity of light driving photosynthesis (actinic radiation) (PAR)—190 µM photons/(m^−2^ s^−1^). The greater changeable fluorescence value (*Fv* = *Fm* − *F_0_*), the maximum photochemical quantum yield of photosystem II (*Fv*/*Fm*), the effective photochemical quantum yield of photosystem II (Y(II)), the electron transport rate, and µmol electrons/(m^−2^ s^−1^) (ETR = Y(II) × PAR × 0.84 × 0.5) were registered for dusky-adapted leaves. Each treatment was concluded with the use of three single leaves as three replications [58].

### 4.7. Determination of Hyperspectral Indices

The data were obtained using hyperspectral imaging (HSI) with the hyperspectral research Module M.Gk. Synergotron hyperspectral camera (Zolotoi Shar, Moscow, Russia). The Normalized Difference Water Index (NDWI) was determined using the following formula, where R is the hyperspectral measurement

NDWI = (R_842_ − R_560_)/(R_840_ − R_560_)

Disease Stress Water Index (DSWI) was determined using the following formula, where R is the hyperspectral measurement

DSWI = R_550_/R_680_

Structure Insensitive Pigment Index (SIPI) was determined using the following formula, where R is the hyperspectral measurement

SIPI = (R_842_ − R_470_)/(R_842_ − R_705_)

### 4.8. Statistical Analysis

Statistical processing of experimental data was performed using parametric Student’s *t*-test and Fisher’s exact tests. Calculations were performed in the Statistica v. 12.0 PL (StatSoft, Tulsa, OK, USA) program. Mean values ± SEM according to ANOVA at α = 0.05 are presented.

## 5. Conclusions

The most susceptible species were *A. hippocastanum*, *A. glabra*, *A. flava*, and *A. × carnea*, while the least susceptible were *A. pavia*, *A. parviflora*, and *A. chinensis*, regardless of crown illumination.

In the leaves of *A. hippocastanum*, *A. glabra*, and *A. flava*, the quantitative content of chl *a* was lowest on the illuminated side, while in the species least affected by leaf miners—*A. pavia*, *A. parviflora*, and *A. chinensis*—this indicator was highest. The opposite situation was observed for chl *b*.

The lowest proline content was observed in species, such as *A. parviflora*, *A. pavia*, and *A. chinensis*, which may be due to greater resistance to stress conditions, which resulted in lower proline synthesis. Impairment of leaf water-retention capacity due to leaf miner feeding is most characteristic of species heavily affected by leaf miners.

Hyperspectral analysis indices, DSWI and SIPI, were also successfully applied to assess injury to horse chestnut trees by the ohrid leaf miner. This opens the prospect of remote damage assessment when monitoring the condition of urban trees.

## Figures and Tables

**Table 1:** Hyperspectral indices reflecting the physiological state of horse chestnut trees.

Species	Side	RGB			Index		
		R	G	B	NDWI	DSWI	SIPI
*A. hippocastanum*	sunny	39.75 ± 1.25	74.25 ± 1.60	37.75 ± 1.11	0.999 ± 0.0012	2.519 ± 0.001	1.250 ± 0.0011
	shady	24.4 ± 2.04	52.4 ± 1.50	24.2 ± 0.73	0.998 ± 0.001	2.629 ± 0.0014	1.222 ± 0.0011
*A. glabra*	sunny	51.8 ± 2.20	101 ± 4.25	35.8 ± 1.46	1.002 ± 0.0013	3.284 ± 0.0012	1.403 ± 0.0011
	shady	46.8 ± 1.91	82.6 ± 1.86	43.6 ± 1.57	1.000 ± 0.001	2.866 ± 0.0013	1.218 ± 0.0011
*A. flava*	sunny	72.6 ± 1.75	113.4 ± 2.77	67.4 ± 2.84	1.000 ± 0.0013	3.191 ± 0.0011	1.372 ± 0.0011
	shady	64.8 ± 4.39	107.6 ± 3.68	55.6 ± 3.43	0.998 ± 0.0012	3.036 ± 0.001	1.383 ± 0.0011
*A. pavia*	sunny	57 ± 1.30	85 ± 2.21	53 ± 2.68	0.997 ± 0.0012	2.973 ± 0.001	1.365 ± 0.0011
	shady	46.2 ± 2.60	70.2 ± 2.78	35.2 ± 2.67	1.001 ± 0.0015	2.416 ± 0.0012	1.248 ± 0.0011
*A. × carnea*	sunny	37.8 ± 1.59	66.4 ± 2.01	36.2 ± 1.46	1.003 ± 0.0013	2.204 ± 0.001	1.157 ± 0.0011
	shady	29.6 ± 1.08	64.6 ± 1.69	24.8 ± 0.66	1.002 ± 0.0012	2.178 ± 0.001	1.211 ± 0.0011
*A. parviflora*	sunny	41.2 ± 2.01	68 ± 2.28	40 ± 1.95	1.000 ± 0.0009	2.004 ± 0.0011	1.156 ± 0.001
	shady	47.2 ± 2.15	74.2 ± 1.32	50.2 ± 1.62	0.998 ± 0.0013	1.888 ± 0.0011	1.133 ± 0.0012
*A. chinensis*	sunny	52.2 ± 5.15	80.4 ± 2.93	37.8 ± 2.24	1.001 ± 0.0011	2.786 ± 0.0012	1.331 ± 0.0011
	shady	54.2 ± 1.85	78.4 ± 1.33	32.6 ± 0.93	0.997 ± 0.0012	3.364 ± 0.001	1.418 ± 0.0011

**Table 2:** Chlorophyll fluorescence indices of leaves of different horse chestnut species under sunlit conditions.

Foliage in the Light	Fv/Fm	NPQ	Y(NPQ)	Y(NO)	ETR
*A. hippocastanum*	0.333 ± 0.0012	0.178 ± 0.0012	0.13 ± 0.0012	0.729 ± 0.0013	11.3 ± 0.3
*A. glabra*	0.491 ± 0.0014	0.173 ± 0.0014	0.149 ± 0.0013	0.754 ± 0.0018	11.5 ± 0.19
*A. flava*	0.371 ± 0.0015	0.244 ± 0.0015	0.153 ± 0.0014	0.703 ± 0.0013	11.5 ± 0.21
*A. pavia*	0.491 ± 0.0011	0.384 ± 0.0013	0.159 ± 0.0013	0.688 ± 0.001	13.5 ± 0.53
*A. × carnea*	0.652 ± 0.0011	0.437 ± 0.0012	0.193 ± 0.001	0.631 ± 0.001	14.8 ± 0.21
*A. parviflora*	0.682 ± 0.001	0.491 ± 0.001	0.198 ± 0.001	0.654 ± 0.001	16.9 ± 0.71
*A. chinensis*	0.717 ± 0.0012	0.521 ± 0.0011	0.326 ± 0.0011	0.589 ± 0.0018	19.1 ± 0.22

**Table 3:** Chlorophyll fluorescence indices of leaves of different horse chestnut species under shading conditions.

Foliage in the Shade	Fv/Fm	NPQ	Y(NPQ)	Y(NO)	ETR
*A. hippocastanum*	0.337 ± 0.0011	0.104 ± 0.0013	0.087 ± 0.0013	0.843 ± 0.0012	5.6 ± 0.23
*A. glabra*	0.441 ± 0.0012	0.149 ± 0.0014	0.117 ± 0.0018	0.701 ± 0.0014	10.1 ± 0.72
*A. flava*	0.358 ± 0.0013	0.219 ± 0.001	0.115 ± 0.0018	0.682 ± 0.001	7.7 ± 0.85
*A. pavia*	0.484 ± 0.0014	0.399 ± 0.001	0.139 ± 0.001	0.663 ± 0.0014	12.8 ± 0.35
*A. × carnea*	0.595 ± 0.0014	0.448 ± 0.0018	0.187 ± 0.001	0.648 ± 0.001	12.8 ± 0.11
*A. parviflora*	0.654 ± 0.0017	0.398 ± 0.0016	0.189 ± 0.0012	0.661 ± 0.0012	11.1 ± 0.43
*A. chinensis*	0.652 ± 0.0018	0.558 ± 0.0012	0.293 ± 0.0013	0.599 ± 0.001	16.3 ± 0.5

## Data Availability

Data are contained within the article.

## Supplementary Materials

The following supporting information can be downloaded at: https://www.mdpi.com/article/10.3390/plants15010086/s1, Table S1: Correlation between Hyperspectral Indices and Chlorophyll Content title; Table S2. Correlation between chestnut species, illumination and physiological and biochemical parameters; Table S3. Correlation between Hyperspectral Indices and Chlorophyll Content; Additional orthodox histograms are presented in the Materials Supplement Figures S1–S9.

## Abbreviations

The following abbreviations are used in this manuscript:

chl *a*	Chlorophyll a
chl *b*	Chlorophyll b
DSWI	Disease Water Stress Index
SIPI	Structure Insensitive Pigment Index
NDWI	Normalized Difference Water Index
F0	Fluorescence value
PAR	The intensity of light driving photosynthesis (actinic radiation)
Fv	The greater changeable fluorescence value
Fm	The maximum fluorescence value
Y(II)	The quantum efficiency of PS II photochemistry
ETR	The electron transport rate
Car	Carotenoids
ROS	Reactive oxygen species
NAD^+^/NADP^+^	Nicotinamide adenine dinucleotide/nicotinamide adenine dinucleotide phosphate
RWC	Water content
FW	Fresh biomass of root or shoot part of seedling
DW	Dry biomass of root or shoot part of seedling
LED	Light-Emitting Diode
Fv/Fm	Maximum quantum efficiency of photosystem II (PSII)
NPQ	The regulated energy dissipation in PS II
Y(NPQ)	The quantum efficiency of regulated energy dissipation in PS II
Y(NO)	The quantum efficiency of non-regulated energy dissipation in PS II
r	Correlation
