# Supplementary material for: Foliar Illumination Affects the Severity of Cameraria ohridella Damage Among Horse Chestnut Species
Source: Plants (Basel). 2025 Dec 27;15(1):86. doi: 10.3390/plants15010086 (PMC12788025; doi:10.3390/plants15010086)
Supplement: Supplementary file 1 [file plants-15-00086-s001.zip › plants-3977647-supplementary.pdf]

Supplementary Materials

Foliar Illumination Affects the Severity of *Cameraria ohridella* Damage among Horse Chestnut Species

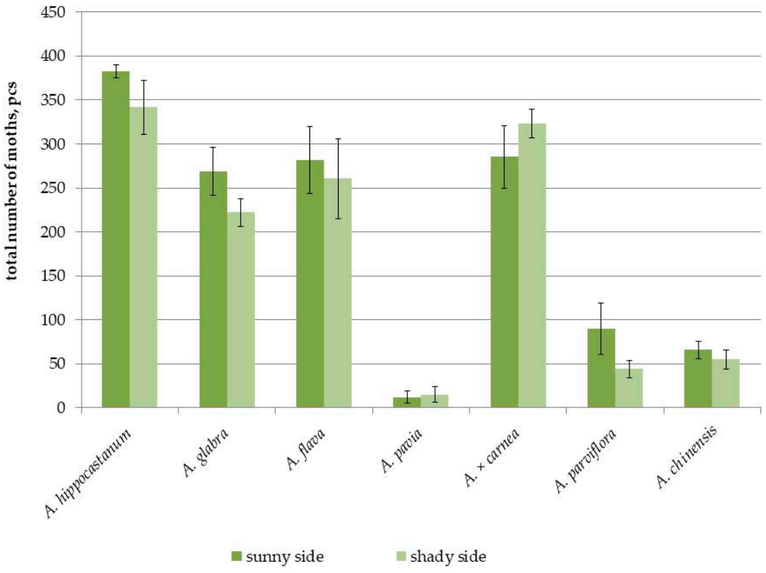

Figure S1. Total abundance of second-generation *C. ohridella* moths on leaves of different horse chestnut species under conditions of varying sunlit illumination. Values are presented as mean ± standard error at $\alpha = 0.05$ according to ANOVA tests.

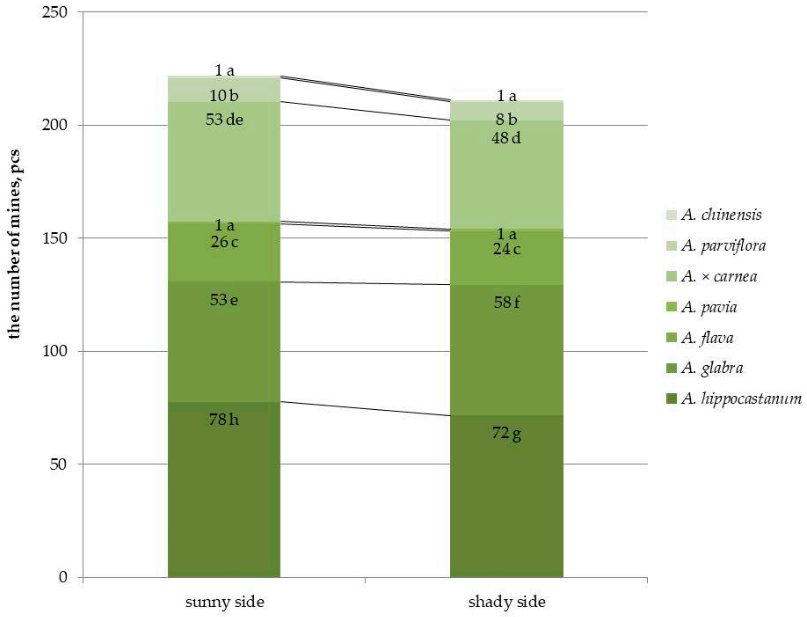

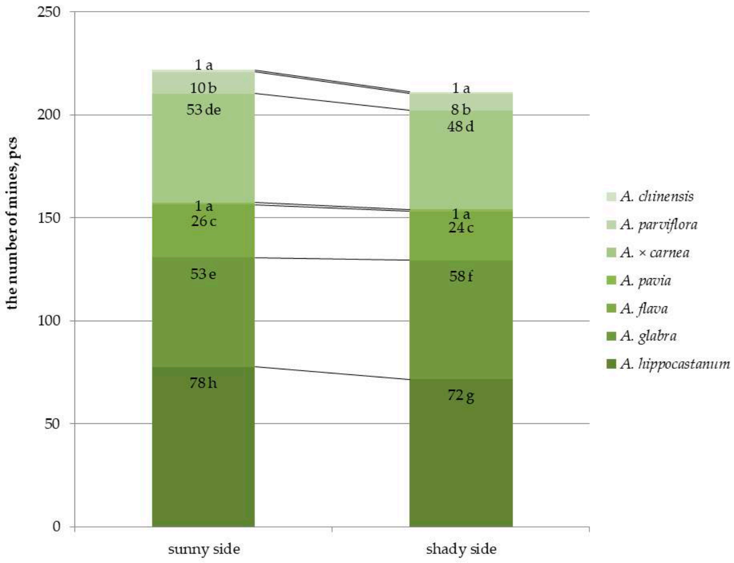

Figure S2. Total abundance of *C. ohridella* mines on leaves of different horse chestnut species, under conditions of varying sunlit illumination. Values are presented as mean ± standard error at $\alpha = 0.05$ according to ANOVA tests.

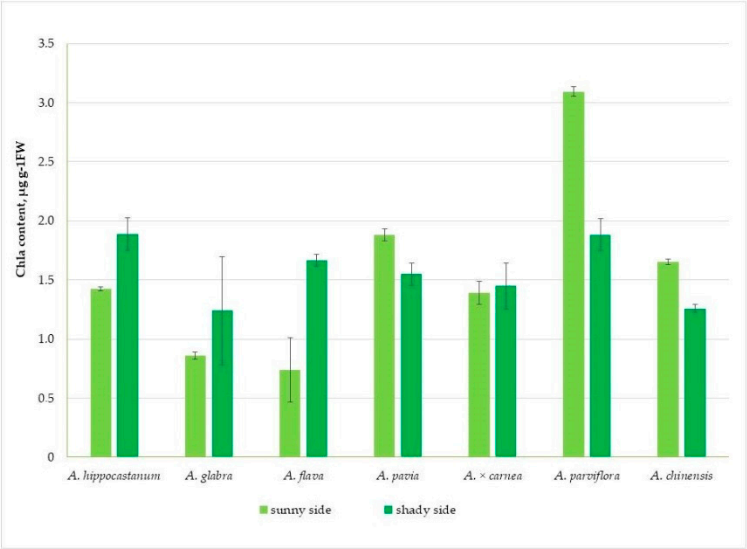

Figure S3. Chlorophyll a (chl a) content in leaves of different horse chestnut species affected by *C. ohridella* under varying foliage illumination conditions. Values are presented as mean ± standard error at $\alpha = 0.05$ according to ANOVA tests.

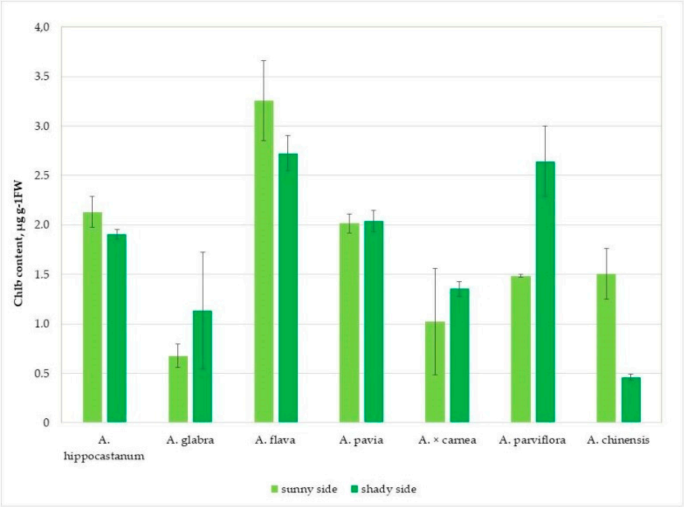

Figure S4. Chlorophyll b (chl b) content in leaves of different horse chestnut species affected by *C. ohridella* under varying foliage illumination conditions. Values are presented as mean ± standard error at $\alpha = 0.05$ according to ANOVA tests.

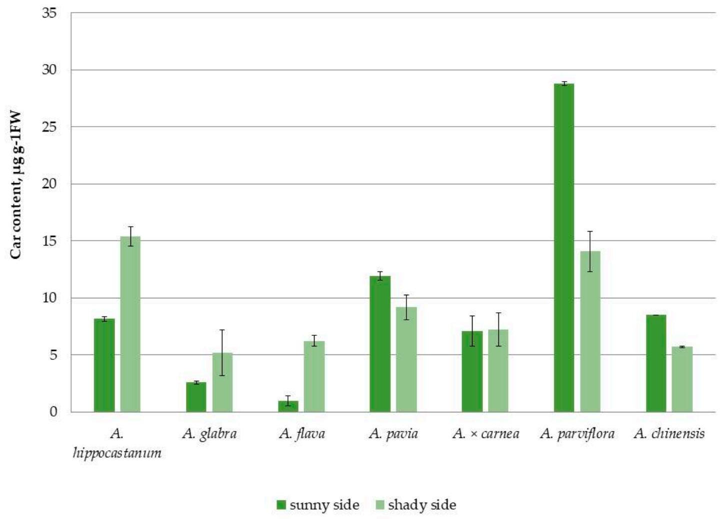

Figure S5. Carotenoid (car) content in leaves of different horse chestnut species affected by *C. ohridella* under varying foliage illumination conditions. Values are presented as mean ± standard error at $\alpha = 0.05$ according to ANOVA tests.

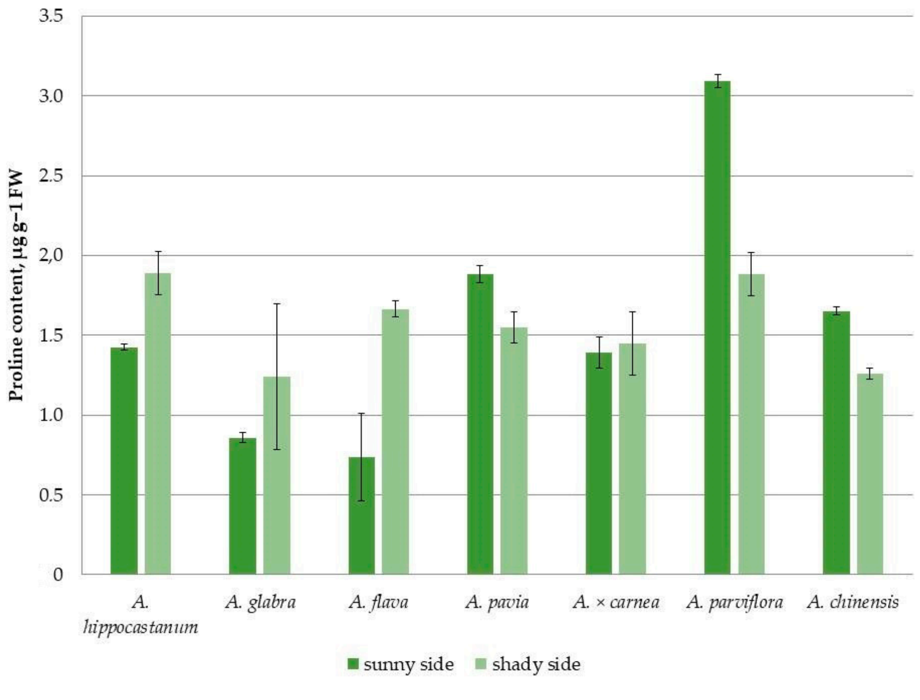

Figure S6. Proline content in leaves of different horse chestnut species affected by *C. ohridella* under varying foliage illumination conditions. Values are presented as mean ± standard error at $\alpha = 0.05$, according to ANOVA tests.

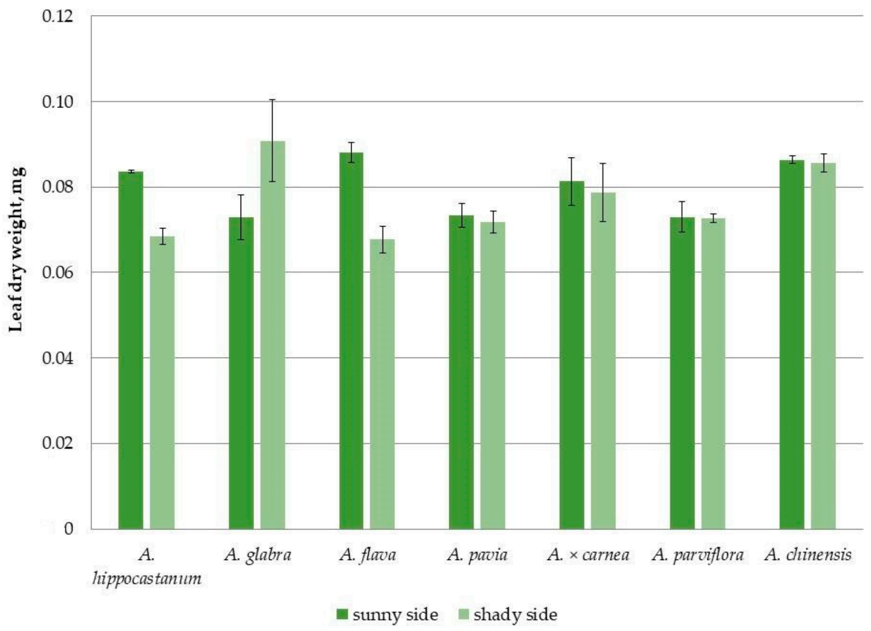

Figure S7. Dry biomass of leaves of different horse chestnut species affected by *C. ohridella* under varying foliage illumination conditions. Values are presented as mean ± standard error at $\alpha = 0.05$ according to ANOVA tests.

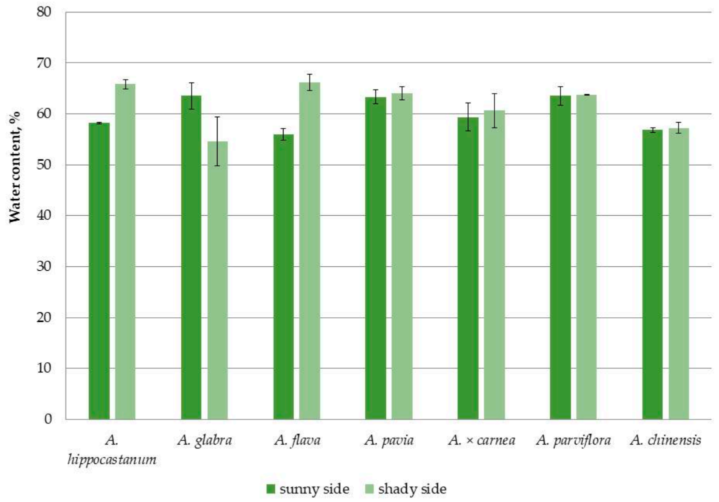

Figure S8. Leaf water content in different horse chestnut species affected by *C. ohridella* under varying foliage illumination conditions. Values are presented as mean ± standard error at $\alpha = 0.05$, according to ANOVA tests.

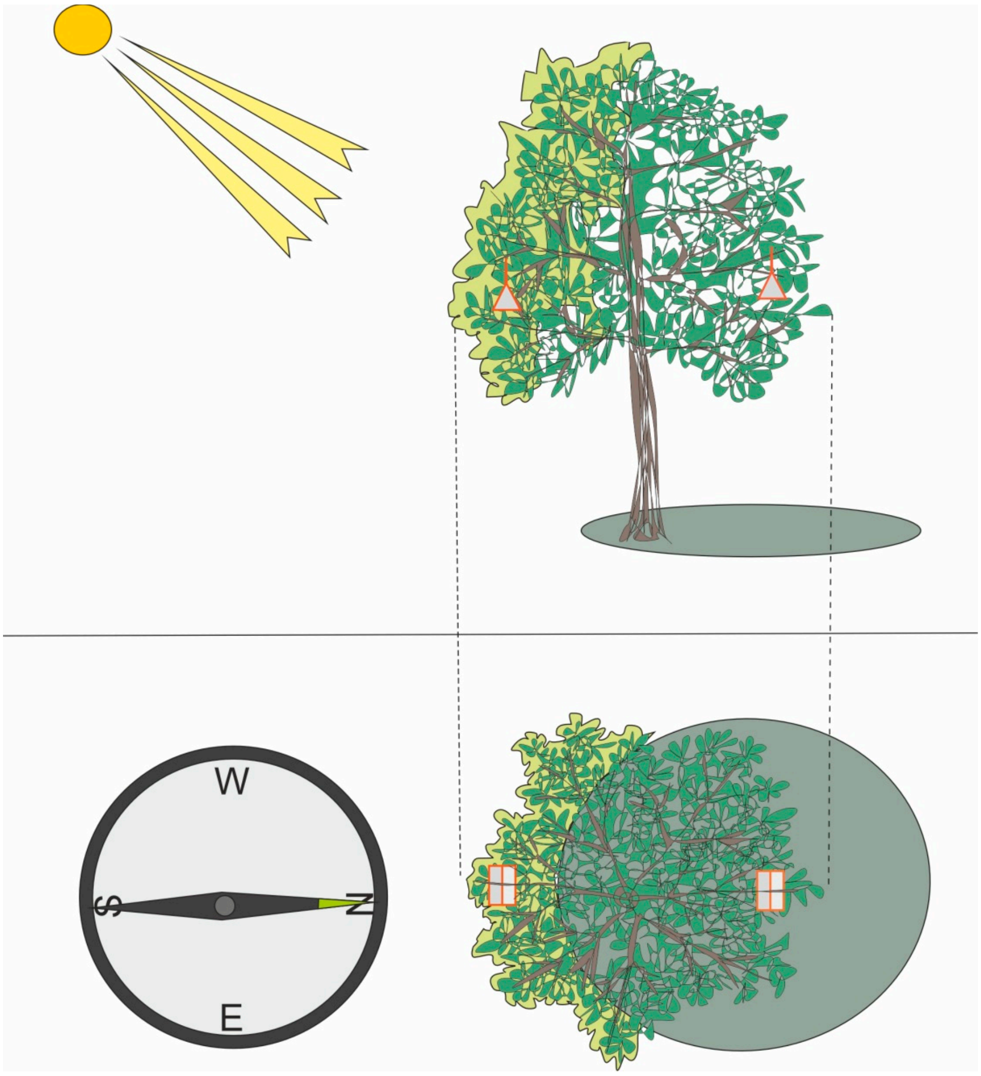

Figure S9. The layout of traps for capturing adult moths of the ohrid leaf miner in crowns of horse chestnut trees depending on sunlight

Table S1. Duncan's test for pairwise comparisons

Cell No.	Species	Crown illumination	{1} 77,667	{2} 71,667	{3} 53,000	{4} 57,667	{5} 25,667	{6} 23,667	{7} 1,0000	{8} 1,0000	{9} 53,000	{10} 48,000	{11} 10,333	{12} 8,3333	{13} 1,0000	{14} ,66667
	Duncan test; variable the number of mines, pcs															
1	*A. hippocastanum*	sunny side		0,010703	0,000033	0,000062	0,000024	0,000020	0,000019	0,000012	0,000055	0,000027	0,000019	0,000018	0,000012	0,000012
2	*A. hippocastanum*	shady side	0,010703		0,000055	0,000142	0,000027	0,000024	0,000018	0,000019	0,000062	0,000033	0,000020	0,000019	0,000012	0,000012
3	*A. glabra*	sunny side	0,000033	0,000055		0,052204	0,000062	0,000055	0,000024	0,000020	1,000000	0,030304	0,000033	0,000027	0,000019	0,000018
4	*A. glabra*	shady side	0,000062	0,000142	0,052204		0,000033	0,000027	0,000019	0,000018	0,042083	0,000292	0,000024	0,000020	0,000019	0,000012
5	*A. flava*	sunny side	0,000024	0,000027	0,000062	0,000033		0,368940	0,000033	0,000027	0,000055	0,000141	0,000062	0,000055	0,000024	0,000020
6	*A. flava*	shady side	0,000020	0,000024	0,000055	0,000027	0,368940		0,000055	0,000033	0,000033	0,000062	0,000142	0,000062	0,000027	0,000024
7	*A. pavia*	sunny side	0,000019	0,000018	0,000024	0,000019	0,000033	0,000055		1,000000	0,000020	0,000027	0,000345	0,002469	1,000000	0,893235
8	*A. pavia*	shady side	0,000012	0,000019	0,000020	0,000018	0,000027	0,000033	1,000000		0,000019	0,000024	0,000414	0,003243	1,000000	0,887735
9	*A. x carnea*	sunny side	0,000055	0,000062	1,000000	0,042083	0,000055	0,000033	0,000020	0,000019		0,038170	0,000027	0,000024	0,000018	0,000019
10	*A. x carnea*	shady side	0,000027	0,000033	0,030304	0,000292	0,000141	0,000062	0,000027	0,000024	0,038170		0,000055	0,000033	0,000020	0,000019
11	*A. parviflora*	sunny side	0,000019	0,000020	0,000033	0,000024	0,000062	0,000142	0,000345	0,000414	0,000027	0,000055		0,368940	0,000475	0,000366
12	*A. parviflora*	shady side	0,000018	0,000019	0,000027	0,000020	0,000055	0,000062	0,002469	0,003243	0,000024	0,000033	0,368940		0,003965	0,003181
13	*A. chinensis*	sunny side	0,000012	0,000012	0,000019	0,000019	0,000024	0,000027	1,000000	1,000000	0,000018	0,000020	0,000475	0,003965		0,880187
14	*A. chinensis*	shady side	0,000012	0,000012	0,000018	0,000012	0,000020	0,000024	0,893235	0,887735	0,000019	0,000019	0,000366	0,003181	0,880187	

Table S2. Correlation between chestnut species, illumination and physiological and biochemical parameters.

Color map of correlations r>= -1 -0,80 -0,60 -0,40 -0,20 0 0,20 0,40 0,60 0,80 1

Variable	Species	Crown illumination	the number of mines, pcs	total number of moths, pcs	Chla content, μg g^{-1}FW	Chlb content, μg g^{-1}FW	Car content, μg g-1FW	Proline content, μg g?1 FW	Leaf dry weight, mg	Water content, %
Species	1,000000	0,000000	-0,749940	-0,713341	0,291293	-0,225283	0,252087	0,033322	0,118518	0,226653
Crown illumination	0,000000	1,000000	-0,027808	0,031860	-0,016965	0,041326	-0,066912	-0,080660	-0,155806	0,165417
the number of mines, pcs	-0,749940	-0,027808	1,000000	0,838214	-0,271513	-0,126915	-0,213750	0,062866	0,090888	-0,182541
total number of moths, pcs	-0,713341	0,031860	0,838214	1,000000	-0,331202	0,103876	-0,316687	0,203097	0,141651	-0,109376
Chla content, μg g^{-1}FW	0,291293	-0,016965	-0,271513	-0,331202	1,000000	0,006288	0,944006	-0,349715	-0,438888	-0,067254
Chlb content, μg g^{-1}FW	-0,225283	0,041326	-0,126915	0,103876	0,006288	1,000000	0,030433	-0,145696	-0,092587	-0,236677
Car content, μg g-1FW	0,252087	-0,066912	-0,213750	-0,316687	0,944006	0,030433	1,000000	-0,363378	-0,402880	-0,064146
Proline content, μg g?1 FW	0,033322	-0,080660	0,062866	0,203097	-0,349715	-0,145696	-0,363378	1,000000	0,256064	-0,093127
Leaf dry weight, mg	0,118518	-0,155806	0,090888	0,141651	-0,438888	-0,092587	-0,402880	0,256064	1,000000	0,043660
Water content, %	0,226653	0,165417	-0,182541	-0,109376	-0,067254	-0,236677	-0,064146	-0,093127	0,043660	1,000000

Table S3. Correlation between Hyperspectral Indices and Chlorophyll Content

Color map of correlations, r>= -1 -0,80 -0,60 -0,40 -0,20 0 0,20 0,40 0,60 0,80 1

Variable	Chl a, sunny side	Chl a, shady side	Chl b, sunny side	Chl b, shady side	NDWI, sunny side	NDWI, shady side	DSWI, sunny side	SIPI, sunny side	SIPI, shady side	DSWI, shady side	Fv/Fm, sunny side	Fv/Fm, shady side	NPQ, sunny side	NPQ, shady side	Y(NPQ), sunny side	Y(NPQ), shady side	Y(NO), sunny side	Y(NO), shady side	ETR, sunny side	ETR, shady side
Chl a, sunny side	1,000000	0,352676	-0,189731	0,256354	-0,405229	-0,251392	-0,723434	-0,602817	-0,500202	-0,644170	0,552340	0,645712	0,661664	0,468851	0,261082	0,370352	-0,419269	-0,208029	0,609698	0,296387
Chl a, shady side	0,352676	1,000000	0,433038	0,695351	-0,445781	-0,216497	-0,387164	-0,382538	-0,325833	-0,415470	-0,280369	-0,201748	-0,087832	-0,251181	-0,354899	-0,334687	0,160825	0,433121	-0,163487	-0,488733
Chl b, sunny side	-0,189731	0,433038	1,000000	0,546242	-0,501290	-0,315981	0,243303	0,231758	0,444678	0,234310	-0,554376	-0,505958	-0,213102	-0,220782	-0,227254	-0,306323	0,130840	0,211413	-0,295301	-0,430323
Chl b, shady side	0,256354	0,695351	0,546242	1,000000	-0,473280	-0,062744	-0,143432	-0,172870	-0,301690	-0,464315	-0,408207	-0,327263	-0,181585	-0,329269	-0,573672	-0,525174	0,332885	0,273842	-0,362860	-0,561532
NDWI, sunny side	-0,405229	-0,445781	-0,501290	-0,473280	1,000000	0,201837	0,002756	-0,110054	0,072499	0,183880	0,351588	0,242057	-0,003466	0,061745	0,242500	0,261063	-0,150703	-0,288278	0,095080	0,194972
NDWI, shady side	-0,251392	-0,216497	-0,315981	-0,062744	0,201837	1,000000	0,031438	-0,063937	-0,364319	-0,403810	-0,037146	-0,061492	-0,101219	0,012690	-0,407235	-0,284498	0,163013	-0,072272	-0,339165	0,097311
DSWI, sunny side	-0,723434	-0,387164	0,243303	-0,143432	0,002756	0,031438	1,000000	0,981522	0,554954	0,726007	-0,506168	-0,566036	-0,560373	-0,376709	-0,180500	-0,304734	0,490439	0,003742	-0,486942	-0,113286
SIPI, sunny	-0,602817	-0,382538	0,231758	-0,172870	-0,110054	-0,063937	0,981522	1,000000	0,569640	0,738882	-0,436338	-0,484121	-0,466383	-0,291941	-0,083951	-0,213342	0,424181	-0,040305	-0,373351	-0,024105

side																				
SIPI, shady side	-0,500202	-0,325833	0,444678	-0,301690	0,072499	-0,364319	0,554954	0,569640	1,000000	0,883482	-0,060995	-0,107878	0,062281	0,228578	0,502840	0,362472	-0,312932	-0,332997	0,165727	0,256303
DSWI, shady side	-0,644170	-0,415470	0,234310	-0,464315	0,183880	-0,403810	0,726007	0,738882	0,883482	1,000000	-0,205070	-0,279032	-0,245539	-0,075500	0,368637	0,188641	-0,001953	-0,091616	-0,028502	0,098287
Fv/Fm, sunny side	0,552340	-0,280369	-0,554376	-0,408207	0,351588	-0,037146	-0,506168	-0,436338	-0,060995	-0,205070	1,000000	0,991779	0,892933	0,865669	0,787491	0,889587	-0,824370	-0,780400	0,913624	0,856256
Fv/Fm, shady side	0,645712	-0,201748	-0,505958	-0,327263	0,242057	-0,061492	-0,566036	-0,484121	-0,107878	-0,279032	0,991779	1,000000	0,927906	0,879293	0,763291	0,873273	-0,832729	-0,757169	0,928933	0,836293
NPQ, sunny side	0,661664	-0,087832	-0,213102	-0,181585	-0,003466	-0,101219	-0,560373	-0,466383	0,062281	-0,245539	0,892933	0,927906	1,000000	0,963650	0,772954	0,871935	-0,930807	-0,766686	0,937559	0,822512
NPQ, shady side	0,468851	-0,251181	-0,220782	-0,329269	0,061745	0,012690	-0,376709	-0,291941	0,228578	-0,075500	0,865669	0,879293	0,963650	1,000000	0,818626	0,900406	-0,941309	-0,843849	0,899861	0,917915
Y(NPQ), sunny side	0,261082	-0,354899	-0,227254	-0,573672	0,242500	-0,407235	-0,180500	-0,083951	0,502840	0,368637	0,787491	0,763291	0,772954	0,818626	1,000000	0,978661	-0,872478	-0,705231	0,912156	0,821311
Y(NPQ), shady side	0,370352	-0,334687	-0,306323	-0,525174	0,261063	-0,284498	-0,304734	-0,213342	0,362472	0,188641	0,889587	0,873273	0,871935	0,900406	0,978661	1,000000	-0,925014	-0,777977	0,960179	0,881195
Y(NO), sunny side	-0,419269	0,160825	0,130840	0,332885	-0,150703	0,163013	0,490439	0,424181	-0,312932	-0,001953	-0,824370	-0,832729	-0,930807	-0,941309	-0,872478	-0,925014	1,000000	0,719070	-0,915978	-0,791193
Y(NO), shady side	-0,208029	0,433121	0,211413	0,273842	-0,288278	-0,072272	0,003742	-0,040305	-0,332997	-0,091616	-0,780400	-0,757169	-0,766686	-0,843849	-0,705231	-0,777977	0,719070	1,000000	-0,709856	-0,874701
ETR, sunny side	0,609698	-0,163487	-0,295301	-0,362860	0,095080	-0,339165	-0,486942	-0,373351	0,165727	-0,028502	0,913624	0,928933	0,937559	0,899861	0,912156	0,960179	-0,915978	-0,709856	1,000000	0,822583
ETR, shady side	0,296387	-0,488733	-0,430323	-0,561532	0,194972	0,097311	-0,113286	-0,024105	0,256303	0,098287	0,856256	0,836293	0,822512	0,917915	0,821311	0,881195	-0,791193	-0,874701	0,822583	1,000000
